# Opioid receptors: single molecule studies shed light on mechanisms of efficacy

**DOI:** 10.1038/s41392-024-01920-2

**Published:** 2024-08-30

**Authors:** Cornelius Krasel, Moritz Bünemann

**Affiliations:** Institute for Pharmacology and Clinical Pharmacy, Philipps-University Marburg, Marburg, Germany

**Keywords:** Drug development, Structural biology

In a study recently published in *Nature*, Zhao et al.^1^ investigate the conformational dynamics of the µ-opioid receptor (µOR). They found evidence for previously unknown conformations of this receptor which could eventually improve the therapy of severe pain.

The µOR is the primary target of opioids, the most potent painkillers available. However, their use is often complicated by undesirable side effects such as respiratory depression and addiction. The µOR is a G-protein coupled receptor (GPCR) that primarily signals through members of the G_i_ family of heterotrimeric G-proteins, although it can also couple to G_15_.^2^ It is also phosphorylated by G-protein-coupled receptor kinases and binds arrestins, particularly when activated by high-efficacy agonists. There has been extensive discussion about the potential therapeutic benefits of biased agonists that would activate only some of these pathways. A deeper understanding of the interaction between the µOR and its ligands could therefore lead to the development of new drugs with fewer side effects.

Zhao et al.^1^ investigated the conformational dynamics of detergent-solubilized µOR using two complementary methods, double electron-electron resonance (DEER) and single-molecule fluorescence resonance energy transfer (smFRET). To label the receptor, the authors employed a cysteine-deficient µOR mutant, allowing them to incorporate cysteines at specific sites of interest and label them with either spin labels (for DEER) or fluorescent dyes (for smFRET). This mutant appears to be somewhat arrestin-biased compared to the wild-type receptor, since it exhibits reduced G protein activation and increased arrestin recruitment, except with the superagonists.

**Fig. 1:**
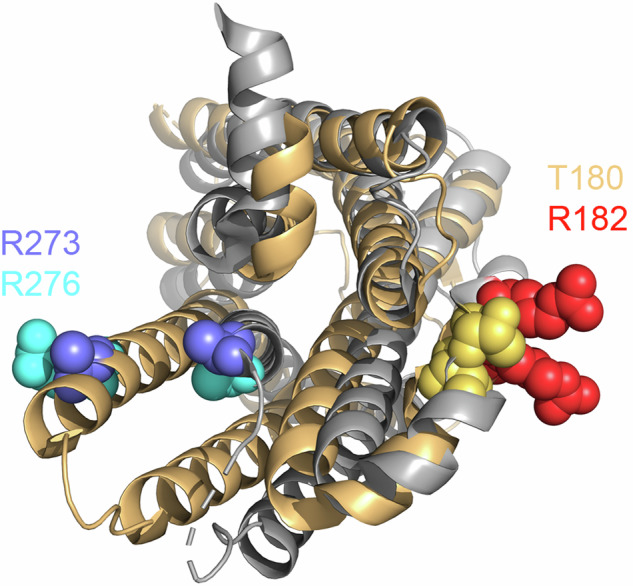
View of an antagonist- (grey, PDB 4DKL) and an agonist- and nanobody-bound (wheat, PDB 5C1M) µOR from the intracellular side. Lysozyme (4DKL) and the nanobody (5C1M) have been removed for clarity. The residues that were labelled in this study for DEER and smFRET experiments are drawn in spheres and labelled

For DEER, protein samples are flash-frozen, thus freezing each receptor molecule in a certain conformation, and investigated by electron paramagnetic resonance spectroscopy at 50 K. The measurable distances within the ensemble are preferably in the 2–5 nm range. The DEER data of the µOR, labelled at positions 182 at the intracellular end of the transmembrane helix (TM) 4 and 276 on TM6, could be explained best by a sum of six distances. Four of these were deemed to be interesting, as they changed upon agonist application and could be matched to high-resolution structures (Fig. 1). Each of these distances represents at least one putative conformation of the µOR. Based on our understanding of GPCR activation, the two shorter distances were assigned to inactive conformations whereas the longer ones were assigned to active conformations. Partial agonists had minimal effect on the basal conformational distribution, and even the full agonist DAMGO forced only a small proportion of the receptor into active conformations. This finding is reminiscent of the first crystal structures of agonist-bound β_2_-adrenergic receptors, which resembled inactive receptors on the intracellular side^3^ unless G-protein or G-protein-mimicking nanobodies were present. It is therefore exciting to see that the superagonists BU72 and lofentanil shifted most receptors to active conformations even in the absence of G-proteins. The equilibrium between the two active conformations further shifted upon G-protein addition, whereas arrestin2 had less influence on the conformational distribution, particularly for partial agonists, regardless of whether they were G-protein-biased (TRV130, PZM21, and MP) or not (buprenorphine). It was previously shown that arrestin interacts poorly with the µOR if these agonists are used. Interestingly, the distance distributions for TRV130, PZM21, MP, buprenorphine, and morphine were quite similar, despite some of the agonists being arrestin-biased. This led the authors to speculate that there might be additional conformational changes that are not detected by DEER. Notably, the existence of four conformations, two active and two inactive, had been previously proposed for the β_2_-adrenergic receptor reconstituted in nanodiscs and labelled with a single fluorescent label.^4^ In that study, the authors could distinguish only two conformations based on fluorescence intensity, but their kinetic data led them to speculate that both the active and inactive state were a sum of two conformations.

smFRET determines the FRET between two fluorophores within a single receptor molecule at room temperature, allowing the receptor to transition between different conformational states during the experiment. The labels for smFRET are bulkier than those for DEER and may therefore report different conformational changes even when attached to the same positions on the µOR. Activation of class A GPCRs such as the µOR in cells occurs typically within tens of milliseconds. However, there may be conformational states that interconvert faster so that the temporal resolution of the smFRET experiments (100 ms) cannot resolve them, resulting in a bimodal FRET distribution. The authors employed two fluorophore pairs, Cy3/Cy5 and Cy3/Cy7, which have different Förster radii (Cy3/Cy5: 5.5 nm; Cy3/Cy7: 4 nm) and thus detect slightly different conformational changes. Due to legal restrictions, they could only investigate a subset of the previously used agonists, and the ligand-free µOR was not stable enough for imaging. Agonists of increasing efficacy caused a decrease in smFRET compared to the antagonist naloxone, consistent with an increase in distance between the two labels. The FRET distribution for the Cy3/Cy7 pair was best fitted as a sum of two Gaussian functions with unchanged centres, indicating an equilibrium of two slowly interconverting distinct conformations, while the FRET distribution for the Cy3/Cy5 pair shifted gradually with efficacy. These results contrast with the DEER data, where most ligands, except the superagonists, did not show major distance changes in the absence of G-protein. The authors speculate that this discrepancy might be caused by small changes in intracellular loop 2 or TM6 not detected by DEER.

The addition of G-protein and arrestin was only performed for the Cy3/Cy5 pair. While arrestin did not have much effect, the addition of G-protein led to the emergence of a second peak with particularly low FRET (indicating a large distance between the fluorophores) which became more populated as the efficacy of the ligands increased. This peak most likely represents the receptor bound to empty G_i_. Interestingly, when GDP was added, this low-FRET peak shifted to slightly higher FRET values, likely reflecting a complex between the µOR and GDP-loaded G_i_. Complexes with GDP-loaded G-protein were previously observed for the β_2_-adrenergic receptor.^5^ The partial agonists TRV130, PZM21 and MP assumed this state already at the lowest GDP concentration tested (0.15 µM) whereas the full agonists DAMGO and BU72 fully assumed this state only at 10 µM GDP. The authors propose that GPCRs occupied by partial agonists are less capable of expelling GDP from the G-protein, which would contribute to the lower efficacy of the partial agonists. The same group has previously made similar observations at the β_2_-adrenergic receptor.^5^
